# Joint Women's V.A.D. Department

**Published:** 1919-10-11

**Authors:** B. C. Oliver

**Affiliations:** Military Department.


### THE EDITOR'S LETTER-BOX.

**[Correspondence on all subjects is invited, but we cannot in any way be responsible for the opinions expressed by our correspondents, who must give their name and address as a guarantee of good faith, but not necessarily for publication. Correspondents are reminded that brevity of style and conciseness of statement greatly facilitate early insertion.]**

### Joint Women's V.A.D. Department.

*To the Editor of* The Hospital.

Sir,—With reference to a letter written by V.A.D. member J. E. Green, headed "The Victimisation of V.A.D.s," which appears in your issue of September 20, I do not think that Miss Green fully understands the financial position of the V.A.D. Nursing and General Service members.

With regard to the nursing members, these ladies sign on at £20 per annum, and at the end of six months receive £22 10s. per annum. They never receive more salary than this, unless they sign for duration of war, when they can rise to £30 per annum at the end of two years' service.

Until last December' their uniform allowance was £5 per annum, but now they receive £8. Their gratuity is £10 for the first twelve months' service, and 10s. for each succeeding month. The utmost gratuity a nursing member may receive is £30 10s. supposing she served for the entire period during which nursing V.A.D. members have been employed in military hospitals. The gratuity is only admissible in respect of completed contracts.

With regard to the General Service members, the lowest rate of pay a member could receive was £22 10s. if she signed for one year. If she signs for duration of war she commences at £26, and in March 1918 the salary of the members who had signed for duration of war was augmented by a bonus of 1s. a week for mobile members.

General Service members receive uniform allowance at the rate of £5 per annum and receive 30s. for the purchase of a great coat on joining.

Any member was eligible for promotion, and on receiving this would be paid at the rate of £40 to £45 per annum. It is probable that the cooks Miss Green refers to, who had in the early days of the war been drawing from £40 to £50 per annum in private service, would have entered as head cooks in military hospitals, drawing £45. This £45 has now been raised to £48.

It would seem that Miss Green is referring especially to members abroad. The unskilled clerk serving abroad receives 32s. 6d. a week, from which a deduction is made of 14s. board, lodging, and laundry, so that she has 18s. 6d. in hand. In addition to this, the General Service Section are entitled to three months' out-of-work donation at 25s. per week if immobile, and six months' out-of-work donation if mobile and having signed a contract for duration of war. Three months' out-of-work donation equalling £16 5s. and six months £32 10s., nursing members are not entitled to this. In addition there is the demobilisation benefit for General Service members; mobile members who sign for duration of war, twenty-eight days' full pay and allowances, and the remainder of the General Service members seven days' full pay and allowances.

I think it will be seen from this comparison that the General Service Section has really no grievance whatever.—Yours truly,

B. C. Oliver, Military Department.

